# It looks like nature - a phenomenological study of the built environment in psychotherapy from psychologists’ and psychiatrists’ perspective

**DOI:** 10.1080/17482631.2024.2408812

**Published:** 2024-10-02

**Authors:** Anne Hagerup, Helle Wijk, Göran Lindahl, Sepideh Olausson

**Affiliations:** a Institute of Health and Care Sciences, Sahlgrenska Academy, University of Gothenburg, Gothenburg, Sweden; b Inland School of Business and Social Sciences, Inland Norway University of Applied Sciences, Inland, Norway; c Department of Quality Strategies, Region Västra Götaland, Sahlgrenska University Hospital, Gothenburg, Sweden; d Department of Architecture and Civil Engineering, Chalmers University of Technology, Gothenburg, Sweden; e Division of Construction Management, Department of Architecture and Civil Engineering, Chalmers University of Technology, Gothenburg, Sweden; f Department of Anesthesiology and Intensive Care/Sahlgrenska, Sahlgrenska University Hospital, Gothenburg, Sweden

**Keywords:** Built environment, indoors environment, outdoors environment, phenomenology, psychotherapy

## Abstract

**Introduction:**

The study aims to examine psychologists’ and psychiatrists’ experiences of built environments, indoors and outdoors, in providing psychotherapy. The research explores how the environment matters in clinical practice from the perspective of psychologists and psychiatrists and seeks to comprehend the significance of the facilities where psychotherapy takes place.

**Methods:**

This study design is explorative and qualitative. Data is generated by eight in-depth interviews with six clinical psychologists and two psychiatrists and was analyzed using an interpretative phenomenological approach.

**Results:**

Our findings revealed that the built environment matters in clinical practice as it appears to be closely linked to fostering a more comprehensive approach and facilitating various associations and themes in psychotherapy. Three superordinate themes emerged from the data: Design as therapeutic tool, Nature as a co-therapist, and lastly, Expanding the therapeutic space, highlights the participants’ perspective on the transformative potential of the built environment to become therapeutic.

**Conclusion:**

The findings reveal how built environments can be actively utilized as tools in psychotherapy. Environments are not to be considered merely as neutral and passive spaces for conducting and receiving psychotherapy rather than experienced as places that may regulate and impact both therapists and patients, the relationship between them.

## Introduction

What is the role of the physical environment in psychotherapy? And what does it mean to transform a room into a therapeutic room? There has been extensive research in clinical psychology concerning therapeutic technique and therapeutic relations between patients and therapists (Norcross & Lambert, 2018). However, little is known about the link between the built environment and its significance for the therapists’ clinical work. Research into how more carefully designed therapy rooms, waiting rooms, and patient rooms could benefit patients, relatives, and staff would make a considerable contribution not only to the care of patients in mental health facilities but also on a societal level, to improved health, increased well-being and more effective disease management (American Psychological Association, 2012; Liddicoat, 2020).

Research emphasizes the impact of a purpose-built physical environment on patient wellbeing and on the working conditions of health care professionals. For example, it is shown that by incorporating design elements, such as access to daylight, access to outdoor spaces, and privacy, it can contribute to a more positive therapeutic experience for the patients (Olausson et al., 2021). Overall, it highlights the importance of creating a supportive physical environment in psychiatric hospitals to enhance patients’ experiences and subsequently possibly promote their recovery. Similarly, when exploring if design in mental health settings could influence anxiety levels, comfort, and therapeutic relationships, the therapists argued for more “natural” surroundings and privacy at the entrances to mental health facilities with a view to enhancing the therapeutic experience (Liddicoat, 2020). Although the research focused on waiting areas in healthcare settings, the researchers suggest examining the applicability of the design suggestions included in their study to different contexts, as the aim was to contribute to an understanding of psychotherapy settings.

In psychotherapy, patients tend to feel that the context is relevant and essential for recovery, in addition to having the possibility to a talk to a therapist who listens and takes their story seriously (Punzi, 2018). In an interview study with clinical psychologists, the participants saw the therapy room as a practical place, but also a welcoming place for relational encounters. The space was important for the capacity of the clinical psychologists to be fully attentive to their patients (Punzi & Singer, 2018). The participants experienced that the room and its interior contained meaning and was a requirement for therapeutic encounters. At the same time, the room became meaningful through the therapeutic relationship and could not be replaced with any random room. The participants emphasized their own need to appreciate the room and feel comfortable, while highlighting that the room is really there for the patients (Punzi & Singer, 2018).

In addition, a critical review of mental health outcome measures in environmental design research identified a strong focus on nature and outdoor environments and concluded that further and more focused research could help develop a knowledge base to support designers in creating spaces that promote mental health (Shin et al., 2021). The study examined environmental correlates in a wide variety of mental health outcomes such as emotions, moods, vitality, executive function, stress, and general well-being. Similarly, another review of mental health facilities indicated that the physical environment is important when it comes to health, well-being, and recovery outcomes of patients (Weber et al., 2022). Physical environments in mental health facilities have incorporated effects from general healthcare settings and patient groups, making it more difficult to adapt mental healthcare settings or transfer patients. For this reason, there is a specific need to bring together evidence of the effects of the physical environment in mental healthcare settings in order to deepen our understanding of how it impacts different psychopathologies (Weber et al., 2022).

The aim of this study was therefore to explore psychologists’ and psychiatrists’ experiences of the built environment in providing psychotherapy.

The research questions that guided this study consists of:

1. What is the role of the built environment in psychotherapy?
2. What does the built environment mean in psychotherapeutic practice?

## Methods

### Theoretical standpoints and concepts

While building on both clinical and environmental psychology, this study focuses specifically on the role of the built environment and its related meanings. The built environment refers to places and surroundings intentionally designed by humans incorporating both indoor and outdoor areas. They often include natural elements such as trees and landscaped areas to represent nature (Joye, 2007).

The built environment is regarded in this study as one with a possible impact on psychotherapy practices. The focus is thus on built environments, indoors and outdoors, and how these two types of physical environments relate in the provision of psychotherapy. The study does not focus on specific artefacts in the design, but rather on how the built environment matters in clinical practice. The research draws on a phenomenological philosophical theory. In phenomenology, the focus is on understanding lifeworld phenomena and their meanings through the experiences they evoke (Finlay, 2011). This perspective emphasizes how these experiences shape an individual’s being and life from their own viewpoint. In the phenomenological framework, the “lifeworld” is understood as the everyday, taken-for-granted world (Finlay, 2011). Our particular interest lies in examining the intentional relationship between therapists and their physical environment in connection with providing therapy. Intentionality in phenomenological research means “directedness of our conscious state” (Moran & Cohen, 2012, p. 167). We aim to explore the underlying meaning structures that constitute therapists’ experiences of this environment and how the environment presents itself to the psychologists and psychiatrists.

Moreover, from a phenomenological perspective, the built environment is understood as both place and space. In this paper, the concepts are used interchangeably and no thorough discussion of them will be offered here. Another concept closely linked to place and space is that of atmosphere. According to Kanyeredzi et al. (2019), atmosphere in regard to mental health facilities is typically studied by examining the relationship between social and environmental factors, and they argue for an approach to atmosphere that places it “in-between” persons and space as a “spatially extended quality of feeling” of which patients are intimately aware.

### Ethical approval and consent procedure

This study adheres to research ethics guidelines as outlined in the Helsinki Declaration. The participants were informed about their right to withdraw from the study without giving an explanation. This information was conveyed both orally and in writing. One participant decided to withdraw before their interview was scheduled. All participants gave written consent to participating in the study and to being individually interviewed. They were also given contact information for the responsible researchers and institutions. To ensure confidentiality and data protection (privacy), the study was approved by the Norwegian Agency for Shared Services in Education and Research (SIKT).

### Design

This study employs a phenomenological approach using qualitative research interviews to generate data offering a greater opportunity to gain detailed knowledge about the phenomenon being investigated (Silverman, 2017). Using a phenomenological approach allows the study to delve into the participants’ own experiences and enter their lifeworld with a genuine will to understand the phenomenon under study, built environments, from the perspective of the psychologists and psychiatrists (Smith & Osborn, 2008).

### Interview setting and intervention

The settings for the interviews were at the Outdoor Care Retreat in Norway. These are two identical wooden cabins located in the parks surrounding two hospitals (hospital A and hospital B). The wooden cabins are located on the hospital premises but separated from the hospital buildings. The wooden cabins are used for psychotherapy. Using the wooden cabins for psychotherapy is regarded as an intervention to “traditional” psychotherapy environments and entails a contextual difference. Standard setting is usually an office with two chairs and/or a sofa where the therapist and the patient are sitting in a 45-degree angle to each other, facing the other person.

Six interviews were conducted in the wooden cabins and two in the therapist’s offices for practical reasons. Hospital A is a university hospital and with 585 beds, in- and out-patient clinics and is mainly a somatic hospital with liaison in psychiatry. The hospital covers 100 000 m2 and has over 24 000 employees at the central and connected sites. Hospital B is a specialist healthcare hospital with over 7000 employees. The hospital consists of both somatic and psychiatric clinics. See photographs 1, 2, 3, and 4 from inside the wooden cabin and the environment outside the cabin. All photographs were taken by the first author.

**Photograph 1:**
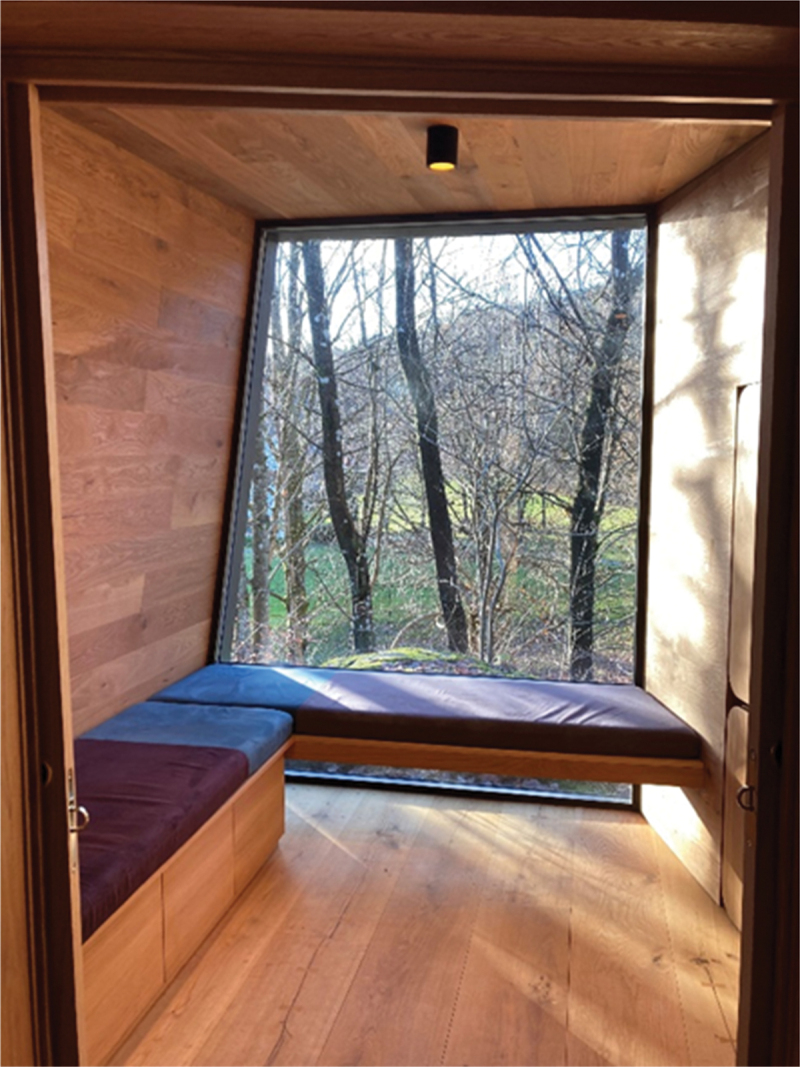
The small conversation room in the wooden cabin used for psychotherapy.

**Photograph 2:**
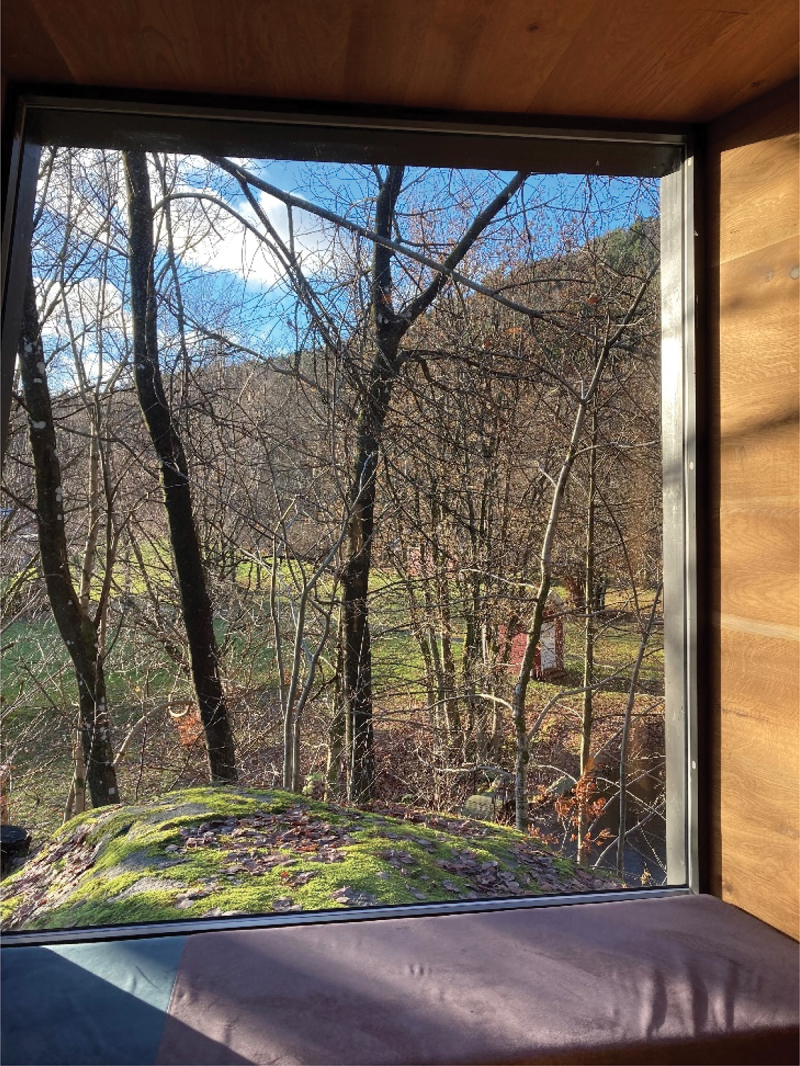
The view to the outdoor environment from the small conversation room.

**Photograph 3:**
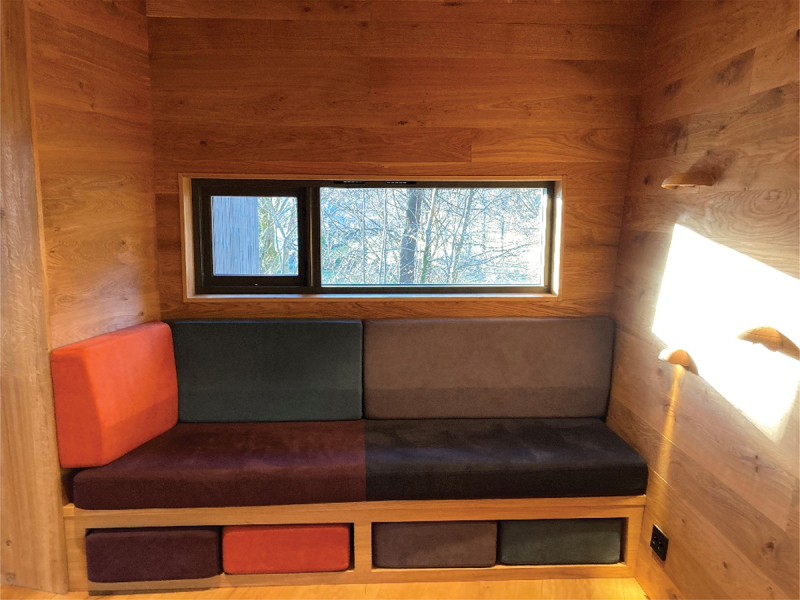
Part of the big main room and window with daylight.

**Photograph 4:**
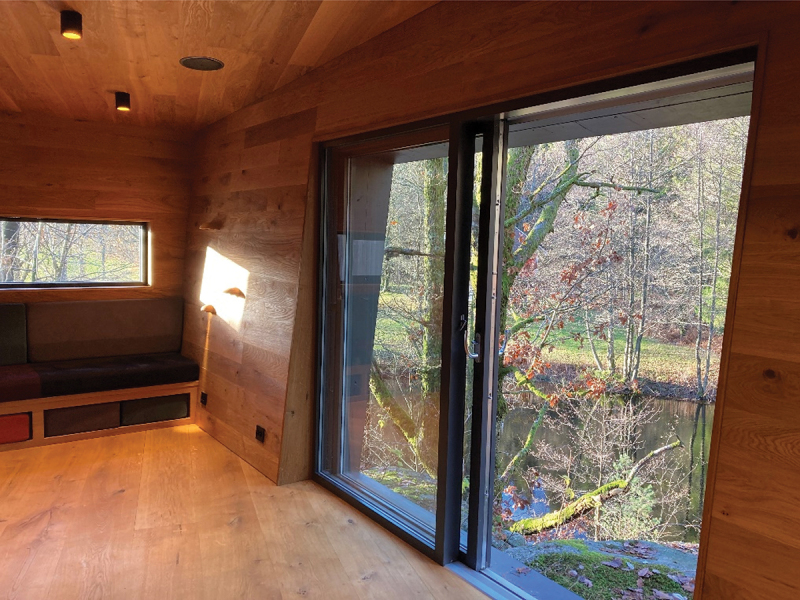
The big main room and its view and access to the outdoor environment used in therapy.

### Recruitment

This study used purposive sampling to recruit participants who could enhance the research findings by providing rich and diverse data (Kvale & Brinkmann, 2019; Silverman, 2017). Two contacts (one at each site/hospital) suggested clinical psychologists and psychiatrists who could be invited to join the study. The suggested participants had either used the wooden cabins when conducting psychotherapy or had been included in the planning of a new mental health facility where nature would be used as an active agent or be built into the facility. The number of participants available for interview was limited, as either having used the wooden cabin for therapy sessions or having experiences with planning of the new physical environment, was a criterion for inclusion. All participants were initially offered the opportunity to be interviewed in the wooden cabin as these were seen as an important setting for the interview.

### Participants

The eight participants in this study comprised seven females and one male between the ages of 25 and 65 years. Two of the participants were clinical psychologists under specialization, four were clinical psychologists with five years of specialization in psychology, and two were medical doctors with five years of specialization in psychiatry. Most of the participants had an extensive experience with clinical work and conducting psychotherapy.

They were initially invited to participate in this study by a contact person. The participants who were invited had first-hand knowledge of using the environments described above in an active way in psychotherapy. The participants could therefore provide rich descriptions and were able to reflect more deeply on the topics investigated in this study.

After the initial invitation, the first author contacted the prospective participants and informed them in detail about what participation in the study would entail. They were given time to consider the invitation. Once the invitations had been accepted, meetings were scheduled.

### Data collection

The first author prepared for the data collection by reflecting on the process, asking “What do I know about this phenomenon?”, “What is known and what is unknown?”. An interview guide was also prepared suggesting a few themes to be explored during the interviews.

The participants were encouraged during the interviews to share their experiences providing psychotherapy in the built environment. The opening questions were: “How do you see coming to the Outdoor Care Retreat affecting patients?” and “How do you experience holding therapy sessions with patients indoors and outside at the Outdoor Care Retreat?”. These were followed by open-ended questions to allow the participants to freely reflect about topics relating to the subject of the study. This provided an opportunity for both the participant and the interviewer to have an active dialogue that could be directed by the participants’ reflections and insights. Key words were sometimes used to elicit the participants’ narrations. During the interviews, notes were also taken as reminders of what had been reflected on and to avoid the repetition of questions.

Follow-up questions were posed to further explore and reveal the meanings of the phenomenon studied. For example, the participants were asked about their experiences relating to the building and its surroundings or what therapeutic possibilities they felt had to be considered when new buildings and surroundings were planned for future psychiatric hospitals. The interviews lasted approximately 1 h, were audio recorded and transcribed verbatim.

### Analysis

The interviews were subjected to interpretative phenomenological analysis (IPA) following Smith and Osborn (2008). IPA combines phenomenology with an interpretive and an ideographical approach which allows the researcher to understand and in detail examine the lifeworld of the participants. This enables for elaborating on the meanings, of the studied phenomenon through the interpretation of capturing the unique and individual experiences. The choice of method for our study was based on the fact that IPA is concerned with detailed examination of personal lived experience (Eatough & Smith, 2017) and seeks to understand the phenomena of interest from a first-person perspective. It believes in the value of subjective knowledge for psychological understanding and seeks to enter the lifeworld of the participant instead of merely analyse it. IPA is initially an inductive process and strives to identify new themes from the available data. At a later stage, it links the findings to relevant theory and empirical evidence through discussion (Smith, 2004). The method of interpretation involves a dynamic movement between the parts and the whole using the hermeneutic circle (Smith & Osborn, 2008).

The process of analysis comprises six steps and guidelines, according to Smith and Osborn (2008), is dependent on the personal analytical work of the researcher which controls the quality of the outcome. The steps involved are: 1. Read and reread the transcribed interview several times and note significant findings in relation to the phenomenon of the study. 2. Start from the beginning to note themes that are becoming evident. 3. Arrange the themes into a more analytical or theoretical order and consider whether they can be grouped together. When free-standing themes begin to be linked to theory, the analysis switches from inductive to deductive. 4. The concepts from the previous steps are transformed into overarching themes. These are then checked against the actual data and themes that lack sufficient supporting evidence can be omitted. 5. The analysis continues with the next interview, and from a phenomenological perspective this means that the process starts again, and the data should speak for itself. Previous interviews must be reviewed if a new overarching theme emerges, and evidence supporting the new theme is sought in all the interviews. 6. The last step involves comparing of all interviews. At this stage, some priorities must be set, and decisions made about which topics are to be pursued further.

The presented suggestions for guidelines in IPA and the step-by-step analysis process were undertaken in the following manner. The transcribed interviews were read several times to identify significant meanings, words, and sentences related to the aim of the study including our guiding research questions. Notes were taken on evident themes while reading the interviews. In the following phase, the text was examined with an open and critical attitude to identify the themes. This was done by marking words or sentences with different colours and then sorting them under corresponding themes. Expressions and psychological terminology that could be used for theoretical comparisons at a later stage were highlighted. Similarly, themes of special interest were selected for deepening and further processing. It was investigated whether there were patterns, common features, or other similarities between the themes. Certain themes were grouped together, overall concepts were clarified, and the themes were sorted into superordinate themes and subordinate themes using mind maps with different colours for the various themes. The participants’ answers were compared with each other and reflected upon. Themes that were in line with the research questions were decided in this step. Please see Table I for details of the analysis process.

**Table I.** Superordinate themes of the role of the physical environment in therapy.

Superordinate Theme	Most Frequent Keywords	Subordinate themes
Design as therapeutic tool	Therapeutic interventions, unique qualities, regulation (i.e., self-regulation and emotion regulation), safe and supportive places, symbolizations, dignity, self-worth, comfort and opportunities, positive distractions, freedom, expansion, trust, creativity, vitality and well-being in addition to unique qualities, natural flexibility, new themes/other themes, and professional or new possibilities, and lastly a few mentioned the key words mutual interplay, connection and positive ambience or related words.	Creating a space for freedom and professional opportunities Intertwining place & space in therapy Stretching the boundaries
Nature as a co-therapist	Different associations, other reflections, impulses and dialogues. Many of the participants were also mentioning metaphors, imagination, unconventionality (or synonyms), self-esteem, experiencing control, coping mechanisms, resources, creating joy or other positive feelings like safe, easier to help, good inner space, spontaneous, accessibility and richer context	Entering a landscape of metaphors
Expanding the therapeutic space	Offering and signaling, (patients’) expectations, vulnerability, challenging the professional role, calming atmosphere, safe to engage, quietness/stillness, tranquility, warm and welcoming, reconnecting (to oneself), ideal place, emotional response, deeper reflection, existential themes, challenging the professional role	Stillness to reflect more deeply

**Figure 1.**
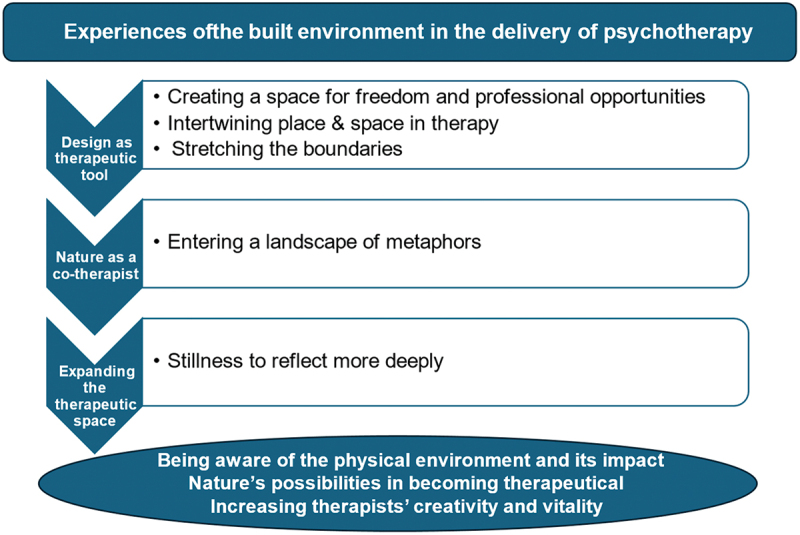
The experiences of the built environment in the delivery of psychotherapy.

To clarify how themes were developed, we were inspired by Shahruddin and Husain (2024) work to explain the analysis process. Please see Figure 1 for a visualization of the participants experiences of the built environment in psychotherapy.

### Rigor

The context of the study has been explained as thoroughly as possible. This is to ensure that readers can judge for themselves the validity, credibility, and transformability of both the analysis and the results (Kvale & Brinkmann, 2019). Reflections such as: “Could this meaning be interpreted in another way?”; “How do we know that this was what the participant meant?”; and “What does it mean?” were discussed critically with the aim of ensuring that the codes and themes are related to meanings in the text. Consolidated criteria for reporting qualitative research (COREQ), a 32-item checklist for interviews (Tong et al., 2007) was used in this analysis. The analysis was conducted by AH in close collaboration with SO and was revised and discussed critically in the research group until consensus was achieved.

### Preunderstanding

Preunderstandings may have influenced the analysis (Kvale & Brinkmann, 2019; Silverman, 2017); however, our respective preunderstandings were systematically challenged through discussions. The different perspectives and experiences of the authors are considered to provide quality assurance in that they reduce the probability of the results are being influenced by the authors’ preunderstandings. Author AH is a clinical psychologist specializing in adult and environmental psychology with extensive clinical experience. Author HW is a professor and registered nurse with long experience of healthcare environmental studies; author GL is a professor and an architect whose research lies in the fields of planning and design of healthcare environments; author SO is an associate professor, a specialist nurse with extensive experience of qualitative research and research into the impact of environment in healthcare settings, including mental healthcare settings.

## Results

### Findings

In exploring psychologists’ and psychiatrists’ experiences of the built environment, we actively sought examples of the role the environment, both built indoors and built outdoors, played in supporting and delivering psychotherapy and how these spaces were used in the therapist’s clinical practice.

The participants’ experiences were related to three superordinate themes, *Design as therapeutic tool* which explores the ways in which the physical space contributes to and influence the participants’ therapeutic work and how therapists utilize the built environment in promoting optimal treatment conditions, *Nature as a co-therapist* is concerned with how nature can be used and can influence psychotherapeutic practices. Finally, *Expanding the therapeutic space* presents the participants’ perspective on the transformative potential of the built environment to become therapeutic.

### Design as therapeutic tool

The physical environment in the context of this study appeared to create a space that encouraged professional possibilities and quality in psychotherapy, in contrast to traditional facilities available for psychotherapy. The therapists had access to a wider range of therapeutic tools to regulate the patient and actively work therapeutically in diverse ways. The surroundings presented themselves as a “sea of opportunities” enabling more creativity and flexibility by offering different frameworks around the psychotherapy. Based on the participants’ reflections, it became apparent that it was helpful for them to acknowledge how they felt the environment could open the way to utilizing new perspectives and spaces in their therapeutic work.

#### Creating a space for freedom and professional opportunities

The therapists reflected that if they were overworked or overstimulated and did not get in touch with their own mental energy (i.e., meaning not achieving access to vitality, excitement, creativity, suggestions for problem solving), it could negatively impact the therapeutic relationship with the patient. Becoming more creative and vital was described as counteracting “tiredness”. There was a striving for freedom and expansion of the space where therapeutic practice could be performed. Freedom meant opening doors to another space both physically and mentally. Furthermore, the participants thought that the way in which the patient is met by the therapist will influence the quality of the therapeutic alliance which in turn may potentially impact the outcome of the therapy. In that sense, one could say that the therapeutic practices were intertwined with the space and the place where the initial meeting took place.

The participants emphasized that in changing the therapeutic environment, the dynamic interplay between therapist and patient also changed and became co-creative in a way that differed from what happens in traditional psychotherapy offices. This could imply a shift or change in therapy and opening the way for other themesfor example, for metaphors to emerge by activating imagination and visualization. The participants were occupied with providing the patient with a voice to counteract self-worth issues. Strengthening the therapeutic interventions by being aware of the built environment’s potential impact implied the existence of a safe and supportive place where the patient was seen and met as the person they were. The built environment that symbolized dignity, warmth and comfort, according to the participants.

I am freer and calmer in my body myself and know that this is a good place to be. And I think this affects how I relate to the patient. I become more playful, smile more and am less tired. And I probably also have more access to creative interventions, or ideas or opportunities that I can suggest. So, the mind is somehow freer. (Participant 4)

#### Intertwining place & space in therapy

The participants emphasized the profound impact the physical setting had in psychotherapy, as it offers unique qualities and opportunities for both closeness and distance, creating a natural flexibility in the therapeutic process. There appears to be a mutual interplay between the therapists’ perception and understanding of the physical space or place, and the therapeutic achievement and intentionality. When therapists see the physical space as a “good place” - one that fosters comfort, trust, and a sense of well-being—it inevitably radiates positivity to their patients. This positive ambience contributes to “in-itself-ness” (being in itself) and meaningful therapeutic experience, promoting a healthier and more productive patient-therapist relationship.

What I am most concerned about is how quickly I get into such a therapeutic relationship here compared to in there [traditional therapy office]. (Participant 4)

The participants also thought that some patients need barriers to “structure the self” and when the environment/room is experienced to be too open they perceive it as threatening and for some patients this may be destabilizing for some of them. The significance of both patients and therapists seeking a deep connection with the physical spaces they inhabit during therapy was also described. The participants emphasized the importance of actively engaging with the environment, wherein carefully chosen objects interact, creating a meaningful dialogue within the space.

The connection between the tangible structure of the physical space and a patient’s inner world emerged as pivotal in the therapeutic process. For instance, when the surroundings symbolize a pleasant and inviting outer space, it was easier for the therapists to foster a sense of self-worth in the patient. Older and outdated environments fail to achieve this in the same explicit manner. This integration of symbolism and environment holds the potential to greatly enhance the therapeutic experience, facilitating an exploration of the patient’s mind and consciousness, and fostering a positive therapeutic relationship.

You have an entrance that is worthy of that person, it must be nice and open and inclusive. (Participant 1)

#### Stretching the boundaries

Using nature-inspired design elements also means bringing nature inside, or taking the therapy outside, allowing for an easier inside-outside relation. All sensory modalities are used, not only the most common, hearing and seeing, smell, feeling the temperature, wind, and sun are all more easily accessible outside than inside. Even therapy practiced in an indoor setting, which provides a view of nature through a window, allows the patients to get access to their imagination more easily and to make use of their senses in a more indirect way. The participants felt that in using the whole body, kinaesthetic senses are involved in a way that differs from when sitting in an ordinary therapy chair.

According to the participants, an environment which is experienced as “nature” and design that made the patients curious, could more easily promote positive distractions, which is about drawing attention outside oneself. In psychotherapy, this may ease the changing of themes, returning to difficult topics, dosages or grading, if and when experienced as necessary by the therapist. A view through a window can distract while simultaneously enabling careful attention and presence. These opposites enable each other. Gazing in the same direction may reduce psychological defence mechanisms and give more access to material that is worked on in therapy.

I feel that they [the patients] realize very quickly that they can set limits here, that things sort of happen on their terms, that there are many opportunities to calm down or sort of distract themselves a little by changing rooms, going out, looking at something … (Participant 6)

The participants reflected on the importance of creating a space to serve as a tranquil oasis amidst the patient’s stress and chaos, offering solace and respite from the storms of stress and anxiety. Many patients feel anxiety and stress which may be activated when they feel threatened. The participants believed that stress and sympathetic activation in the nervous system was important in therapying order to access the patient’s ability to mentalize and use cognition. Also, enabling the patients to experience control may reduce stress and activation. The participants were thought that nature and nature-inspired design could reduce the experience of stress.

### Nature as a co-therapist

Closeness to nature may give rise to different associations/themes in psychotherapy by providing a richer context that could contribute to other reflections, impulses, and dialogues. The participants stressed the importance of having surroundings that engage the imagination. Being creative was made easier by nature-inspired design and the outdoor made it easier to be creative in producing responses that led to more inventive solutions and to alleviating problems and emotional pain when needed. A therapeutic perspective that focuses on creativity and unconventionality, in the form of being open to new associations and themes, being spontaneous, creating joy and laughter, may be mitigate what is perceived as painful for the patient.

#### Entering a landscape of metaphors

The physical surroundings were perceived as a metaphor for an inner dimension. The therapists were concerned with creating a good inner space, which is difficult for some patients because of their negative experiences and low self-esteem. The physical surroundings may be perceived as a good outer space. Nature can also act as a physical symbol of what was worked on in therapy and thus provide a lot of metaphors for use in therapy. For example, different times of the day, daylight or darkness, daily rhythms, seasons, wind, a river, a storm, or a tree could serve as a metaphor for the self or the patient’s current situation. Physical symbols provide easier access to imagination and visualization. There are two dimensions—the invisible and the visible—existing at the same time and referring to different levels in the therapy. Externalizing the inner and invisible dimension to an outer visible dimension through seeing and processing makes the therapy themes that are being worked on more accessible.

Going outside into nature or looking out a of window could ease emotion regulation for patients by letting them find their own ways in which to feel good about themselves. Having access to more flexibility, and something pleasant to look at (like nature), makes it easier to help the patients to regulate their emotions, according to the participants. Experiencing control and feeling safe may, in addition, help patients to be more amenable to psychotherapy. If they experience the surroundings as warm and welcoming, it helps them both to feel safe at a psychological level and to gain easier access to resources and coping mechanisms. The physical surroundings made it possible for more resources to emerge by easing emotion regulation.

We are concerned with creating an inner good place, and for some [patients]this is difficult and then this [the building and the surroundings] becomes an external good place. That you know exists. (Participant 6)

### Expanding the therapeutic space

The participants also reflected on what makes an “ordinary” environment therapeutic, i.e., the physical room and what it offers and signals to patients and to therapists and the kind of work being done there. They were also concerned about the expectations the patients have when entering the room/space. Is this a safe place for the patient to unveil problematic issues to be worked on in therapy? Is it safe to be vulnerable, to reveal sides of themselves that the patient does not usually disclose.

Because there is something about that room in itself and what it invites, but I think that is also an important factor … (Participant 8)

The atmosphere for was perceived by the therapists to be essential for psychotherapy. For example, a calming atmosphere should permeate the whole room so that it can become a sanctuary—a place for reconnecting to oneself. It should also engender a feeling that is safe to engage in conversations that may be difficult in therapy. A warm and welcoming atmosphere surrounded by nature and nature-inspired elements, big windows, daylight, the use of wood, amongst other things, were perceived as making the built environment feel like an ideal place for psychotherapeutic practice. Sensing the atmosphere may increase the accessibility to emotional responses that may be worked on in therapy. Negative sounds, flickering light, stressful movements or unfriendly touches are often perceived as burdensome.

#### Stillness to reflect more deeply

According to the participants, environments may be noisy. The surroundings are quiet and because the sound level is low and they are surrounded by nature, there is a feeling of calmness. The therapists felt that the stillness provided opportunities for deeper reflection, that the pace in the therapeutic conversations was calmer and there was more tranquillity. The nature that surrounded the premises provided these opportunities. The therapists thought that the nature-inspired design and the calmness were essential, particularly if the conversation included existential themes.

And then it’s much easier to be quiet here. So, you can just sit for a long time and just look and have long breaks, look at nature. And I think that also has something to do with the pace of the conversation. And maybe also the themes then. (Participant 7)

There was also a consciousness amongst the therapists that nature belongs to everyone, while a traditional psychotherapy office is usually intuitively seen as belonging to the therapist. A more open environment may lead to the therapeutic professional role being challenged, including presumptions about who does what and how to relate. The participants emphasized the importance of being aware of this.

## Discussion

In this study, we aimed to explore psychologists’ and psychiatrists’ experiences of the built environment when providing psychotherapy. Our findings reveal that, for the therapists, the physical environment provided more nuances for therapeutic work and expanded the therapeutic space. The three superordinate themes explore how the physical environment contributes to and influences therapeutic practice. Physical environments could enable and influence practice by offering different frameworks around the psychotherapy.

A notable finding in this study is that the built environment created a space for professional possibilities and qualities in psychotherapy. They provided a wider range of therapeutic tools which traditional psychotherapy settings could not offer in the same way. The findings suggest that built environments could give rise to different associations/themes in psychotherapy by providing a richer and more nuanced context that may contribute to the introductions of other reflections, impulses, and dialogues into the therapeutic encounter.

The participants consisted of psychologists and psychiatrists and had various interpretations of built environments, both indoors and outdoors. They used words like natural, natural design and nature. The authors have tried to categorize the various terms within built environments, either indoors or outdoors, or both.

### Being aware of the built environment and its impact

The participants in this study reflected on what constituted an “ordinary” therapeutic environment. At the same time, they are interested in how to strengthen therapeutic interventions, expand the therapeutic space, and how design could be used as a therapeutic tool. The participants felt that their awareness of the role of the built environment in supporting the patient in the therapeutic work and their active use of existing possibilities might enable them to offer the patients supportive and comforting environments.

Mental health facilities and psychotherapy offices can be experienced as stressful and according to Punzi (2018), the experience of the context is relevant for recovery. In her study, clinical psychologists experienced the therapy room as a practical place, but also a welcoming place for relational encounters. The space was essential to the clinical psychologists’ capacity to be fully attentive to their patients (Punzi & Singer, 2018). The room and its interior carried meaning and were important for therapeutic encounters. Simultaneously, the room became meaningful through the relationship between the therapists and their patients and could not be replaced with any other room.

In our findings, the participants put forward the importance of being able to regulate themselves and the patients, and that this was made easier in environments which they experienced as supportive. Ulrich’s theory of supportive design (Ulrich, 1991) provides a broad conceptualization of the ways in which the environment in healthcare can increase stress. Research shows that the so-called psychologically supportive environments reduce stress (Andrade & Devlin, 2015; Ulrich, 1991; Ulrich et al., 1991). Individuals have different levels of mental capacity available at different points in time. Being able to offer built environments that reduce stress and therefore enhance mental capacity and to offer the individual various physical stimuli to help them manage and cope improves access to psychotherapeutic treatment.

The built indoor and outdoor environments in this study, seem to offer the potential to provide increased flexibility and a more freely regulated level of stimuli than a more traditional psychotherapy office. In such an environment, the therapist normally has less possibility to vary the level of stimuli for individuals at different times in the recovery period. We understand these findings as related to stimuli regulation, using the Triangle of supportive environments (Bengtsson & Grahn, 2014), because they can be related to or incorporated into, their model and be understood even if the physical environment is (partly) indoors.

The model illustrates an individual’s relationship with physical and social environments and how this is connected to their executive functions (Bengtsson, 2015) and to what degree this has relevance when conducting psychotherapy. The triangle was originally developed for outdoor environments but can be applied to a wider context, both indoor environment and outdoor planned and unplanned environments. According to Bengtsson (2015) individuals with stress-related disorders at the bottom of the triangle experience low well-being and high sensitivity to the environment. Directed inwards engagement is usually managed and these individuals need environments where they can be alone. The bottom of the triangle is where the need for environmental support is highest. The triangle’s second level comprises individuals who can manage emotional engagement. The individual wants to be alone but wants visual contact with others from a distance. The third level is where individuals engage in social and active environments and take part in group activities. At the fourth-level individuals exhibit outward engagement as they have achieved high levels of executive function and can easily manage social and active environments.

The participants in this study spoke of giving the patient a voice to counteract negative aspects of self-worth issues. In their opinion, when the surroundings signify a pleasant and inviting outer space, a sense of self-worth is fostered in the patient which may support and improve their self-image. The results from a study (*n* = 170) of the role of self-image as a predictor of psychotherapy outcome suggest that improvement in self-image may be important for a good outcome in psychotherapy (Ryum et al., 2015).

The participants experienced being less stressed and became more vital and creative when psychotherapy was conducted outside the ordinary therapy office. This finding is in line with a literature review of the role of healthcare facility design on the mental health of health care professionals (Jin et al., 2023). As stress and mental exhaustion among healthcare professionals is becoming more evident, research shows that it is significantly reduced, and even prevented, by adapting the physical design of healthcare facilities, removing environmental stressors and providing restorative experiences. Ongoing research indicates that the connection between the indoor and outdoor in healthcare environments should be incorporated in their design, that they should be considered as healing places for all their users and that the design must be oriented towards counteracting stress (Karanikola et al., 2020).

The participants observed how the built environment had an impact on the therapeutic relationship. The theory of “the holding environment” (Winnicott, 1971/2005) originally described that quality of motherhood perceived by the infant as an environment capable of providing physical and emotional support. The holding environment/the mother holds the infant, both literally and figuratively, with focused attention and genuine concern and provides comfort. In the context of therapeutic work, it expresses a psychological space and a therapeutic alliance that is not only safe and supportive but also offers privacy and protection. “A holding environment” in therapeutic practice may be experiences through focusing on providing a supportive setting. In this study, the participants were concerned with how the meeting between the patient and the therapist could influence the quality of the therapeutic alliance and therefore potentially the quality and outcome of therapy. They were also insightful about making intentional adjustment in the therapeutic environment which change the interaction between the therapist and the patient. The interaction becomes co-creative in a different way and a safe and supportive place is established where the patient is seen and met for the person they are.

### Nature’s possibilities in becoming therapeutical

The effects of nature on health outcomes are well documented (Adams et al., 2014; Jordan, 2015). The Norwegian Government has encouraged hospitals to use nature in treatment for mental disorders (2017) because of the positive impact it can have on physical and psychological health. Although nature and the environment have historically been valued as healing and supportive, and it is hospitals and institutions in Norway today that emphasize this, most of the therapy nevertheless takes place indoors in office premises, without thinking about the potential effects of the surroundings. Rarely are building design and natural surroundings used intentionally as a tool in the therapeutic process. There is now, however, increasing focus and research on the influence of building design and environment on therapy and treatment in mental health buildings (Weber et al., 2022).

The participants in this study reflected on the therapeutic impact of nature and “natural” materials. This is in line with a study on the influence of the “natural environments” on creativity (Yeh et al., 2022). Emotion regulation was also promoted as the patients could more easily experience their own way of feeling good about themselves. These findings are also presented in a study on the active use of natural environments for emotion regulation (Johnsen & Rydstedt, 2013) and nature’s potentially positive influences in psychiatric spaces are discussed in another study (Simonsen et al., 2024).

### Increasing therapists’ creativity and vitality

The participants in this study emphasized that becoming more creative and vital, and counteracting being tired, was easier in the wooden cabin and its surroundings. Being mindfully present was more difficult if the therapist was overstimulated. When they experienced more positive sensations in and around the wooden cabin, they felt more connected to themselves and the patient. Psychologists and psychiatrists are at risk of burnout due to repeated exposure to the strong emotions and distress of their patients. A qualitative systematic review and meta-synthesis has provided an overview of the experience and impact of burnout in psychological therapists (Vivolo et al., 2024). The authors have focused on strategies to reduce or avoid burnout and identified implementing self-care strategies as a possible way of reducing or preventing it. These findings are in line with those in the present study, showing that taking breaks, reducing the pace and gazing into nature, in addition to being in purpose-built physical environments may counteract tiredness by reducing the overstimulation that strong emotions and distress can increase. A literature review on the role of nature in coping with psycho-physiological stress shows a strong relationship between exposure to nature and recovery from physiological stress and mental fatigue (Berto, 2014).

### Methodological strengths and limitations

The phenomenological study provided insight into clinical psychologists’ and psychiatrists’ experiences of the relationship and the connection between the physical environment and psychotherapeutic practice. Using qualitative interviews allowed a broader opportunity to access detailed insights into the phenomenon investigated (Silverman, 2017). By using a phenomenological approach, the researcher had the possibility to research the phenomenon in depth and to enter the participants’ psychological world (Smith & Osborn, 2008). By attending to convergence and divergence, close analytic reading of participants’ words, tying to develop a vigorous experiential and/or existential account, and lastly our endeavour to construct a compelling, unfolding narrative, we have sought to achieve an IPA of high quality (Nizza et al., 2021).

The researchers have carefully considered that, while high reliability regarding the interview findings is desirable to counteract arbitrary subjectivity, focusing on reliability too strongly can counteract creative thinking and variation (Kvale & Brinkmann, 2019). Furthermore, when an interviewer can use their own interview style, improvise and follow their senses, it tends to provide better conditions for the results to emerge. According to Kvale and Brinkmann (2019) validity has to permeate the entire research process and we have tried to keep this in mind through planning, interviewing, transcribing, validating and reporting. Also, by interviewing experts in their field (psychologists and psychiatrists), one strength may be the access to a meta-perspective about themes and experiences related to the phenomena of interest (Kvale & Brinkmann, 2019). In this research, we have focused on being rigorous and transparently trustworthy with the intention to enriching the work of practitioners (Finlay, 2011). We have applied the 4 R’s *rigor*, *relevance*, *resonance*, and *reflexivity* according to Finlay (2011) when discussing and evaluating the research within the team.

A limitation that may have had an impact on the answers the participants gave, is that they were mainly positive about the physical environment, possibly because it was new and represented a different approach to therapy. They may therefore have been prejudiced and the fact that they were allowed to be in the “new” environment could be a bias. They also knew the researcher was positive and especially interested in the topic under investigation. The first author [interviewer] was aware of this phenomenon and tried to minimize the risk by constantly reminding herself of it and at a later stage discussing the phenomenon with the other researchers/authors. The results can be influenced by the interaction between researcher and participant and by the fact that the participants’ experiences are subject to the researchers’ interpretation and different researchers may arrive at different results (Willig, 2013). There were no previous personal or professional relationship between the researcher and the participants.

We did not conduct a pilot interview as it was impractical at the time the study was conducted, and this was another challenge. Pilot testing could have made the questions more pertinent to the research and clearer. The sample was a strategic sample based on an availability principle. It was relatively small due to a lack of participants that were either clinical psychologist or psychiatrist and had experience with the new therapy environment. The participants comprised one male and seven females of various ages. They differed in their experience related to conducting psychotherapy and this may have influenced their answers. If the sample size is too small, generalization is made more difficult, but on the other hand, if the sample size is too big, carrying out a more comprehensive in-depth analysis is more time-consuming.

How did the physical environment influence the answers the participants gave in this study? Two interviews were conducted in an ordinary psychotherapy office and six interviews in the wooden cabin. Analysis of the interviews does not seem to reveal any major differences in the answers. However, the two groups of participants differed in their experiences because they worked in different locations (hospital A and hospital B) so the data provided by the interviews might also have given richer data and thus perceived as more interesting. If the physical environment influenced the activity (usually psychotherapy, but in this case, research interviews), there should also be some nuances in the way the interview’s physical environment was experienced. The reason for lack of differences in the answers relating to this, might be that the number of interviews is small and difficult to compare. It could also be that the participants were familiar with the physical environments, even if they were not in them at the time of the interview, and they were aware of how they experienced being there. Furthermore, there were seven women and only one man, which could have influenced the answers assuming that different genders have different values or reflections. A question this study does not answer is “Do clinical psychologists and psychiatrists differ in their meanings due to different backgrounds and education?” which must be left to further research.

## Conclusion

The results of this study revealed how the physical environment can be utilized as an active tool in psychotherapy. This means that the built environment, both indoors and outdoors should not be considered merely as neutral and passive places for conducting and receiving psychotherapy, but rather as an active therapeutic tool that may regulate and impact therapists, patients, the relationship between them, and therefore, potentially the quality of the therapy and therapeutic outcome. By being open to the idea that the physical environment in psychotherapy may be influential, potentially makes a more beneficial therapeutic outcome possible.
